# Ten years of the International Patient Decision Aid Standards Collaboration: evolution of the core dimensions for assessing the quality of patient decision aids

**DOI:** 10.1186/1472-6947-13-S2-S1

**Published:** 2013-11-29

**Authors:** Robert J Volk, Hilary Llewellyn-Thomas, Dawn Stacey, Glyn Elwyn

**Affiliations:** 1 Department of General Internal Medicine, The University of Texas MD Anderson Cancer Center, 1515 Holcombe Blvd., Houston, Texas 77230 USA; 2 Department of Community and Family Medicine, The Geisel School of Medicine at Dartmouth, Hanover, NH 03755 USA; 3 The Dartmouth Institute for Health Policy and Clinical Practice, The Geisel School of Medicine at Dartmouth, Hanover, NH 03755 USA; 4 School of Nursing, University of Ottawa, 451 Smyth Road (RGN Room 1118), Ottawa, Ontario, K1H 8M5, Canada; 5 Clinical Epidemiology Program, Ottawa Hospital Research Institute, 501 Smyth Road, Box 201B, Ottawa, Ontario K1H 8L6, Canada; 6 Cochrane Institute of Primary Care and Public Health, Cardiff University School of Medicine, Health Park, CF14 4YS, UK

## Abstract

In 2003, the International Patient Decision Aid Standards (IPDAS) Collaboration was established to enhance the quality and effectiveness of patient decision aids by establishing an evidence-informed framework for improving their content, development, implementation, and evaluation. Over this 10 year period, the Collaboration has established: a) the background document on 12 core dimensions to inform the original modified Delphi process to establish the IPDAS checklist (74 items); b) the valid and reliable IPDAS instrument (47 items); and c) the IPDAS qualifying (6 items), certifying (6 items + 4 items for screening), and quality criteria (28 items). The objective of this paper is to describe the evolution of the IPDAS Collaboration and discuss the standardized process used to update the background documents on the theoretical rationales, evidence and emerging issues underlying the 12 core dimensions for assessing the quality of patient decision aids.

## Background

### Patient decision aids

Patient decision aids are tools designed to help people participate in decision making about health care options, with the goal of promoting deliberation between patients, health care providers, and others about those options. They provide information about the options, and help patients to construct, clarify, and communicate the personal values they associate with the different features of the options. Patient decision aids do not advise people to choose one option over another, nor are they meant to replace practitioner consultation. Instead, they provide structured guidance in the steps of decision making and to prepare patients to make informed, values-based decisions with their practitioner [1-3].

### The International Patient Decision Aid Standards (IPDAS) Collaboration

The International Patient Decision Aid Standards (IPDAS) Collaboration is a world-wide group of researchers, practitioners, and stakeholders who are interested in the design and use of patient decision aids. It was established in September, 2003, at the International Shared Decision Making (ISDM) Conference in Swansea, Wales. (“Shared Decision Making”, or SDM, is defined as an approach where clinicians and patients make decisions together using the best available evidence. Shared decision making emphasizes respect for patient autonomy and promotes patient engagement, by encouraging patients to think about the available screening or treatment options and the likely benefits and harms of each option in preparation for communicating their preferences and selecting the best course of action for them. Patient decision aids are often used during this process of shared decision making [4,5].)

The *overall* purpose of the IPDAS Collaboration is to enhance the quality and effectiveness of patient decision aids, by establishing an evidence-informed framework for improving their content, development, implementation, and evaluation. As its *initial* task, in 2003, the IPDAS Collaboration undertook the identification of internationally-approved set of standards that could be used by individuals and organizations to guide: a) the development new patient decision aids; as well as b) the evaluation of the quality of existing patient decision aids.

### Why are standards needed?

At the 2003 ISDM Conference, the IPDAS Collaboration agreed that a problem may be emerging. Hundreds of patient decision aids were available or were being developed by many different individuals and groups around the world. The growth of the internet and mobile technologies provided a tremendous opportunity for dissemination of patient decision aids. However, people may have difficulty knowing whether or not a patient decision aid is a source of reliable health information that can help in decision making. Therefore, the need for a set of standard criteria was identified as a mechanism to guide quality appraisal of patient decision aids by a wide variety of individuals and organizations that use and/or develop patient decision aids.

## Processes and products

### 2003-2006: Developing the IPDAS checklist

The IPDAS Collaboration’s Steering Group led this initial task; see Elwyn et al. for the procedural details [6]. To begin, the Steering Group sought opinions from the participants in the 2003 ISDM Meeting and from the shared decision making electronic listserve (“SMDM-L”) about which broad evaluative dimensions should be used for assessing the quality of patient decision aids. Twelve such quality dimensions were identified.

From there, 12 workgroups were formed. Each workgroup was assigned one of the quality dimensions and was given the following tasks: 1) offer a definition of that quality dimension; 2) outline the theoretical rationale for considering that dimension as an important aspect of the quality of patient decision aids; 3) provide a summary of the relevant evidence base underlying that quality dimension; and 4) list the relevant theoretical and empirical references for that dimension. The IPDAS Collaboration’s resulting 12-chapter “Original Background Document” was published in 2005; it can be found at the IPDAS collaboration website (http://ipdas.ohri.ca/resources.html). Of the 12 chapters, three were subsequently published as peer-reviewed manuscripts focused on providing information, measuring decision quality, and communicating probabilities [7-9].

The Steering Group envisioned a checklist of standard criteria reflecting these 12 key evaluative dimensions. Therefore, during this initial phase, each workgroup was also asked to propose and draft the specific evaluative criteria that they thought should be used to gauge whether or not a patient decision aid satisfactorily addressed their quality dimension.

Next, a modified Delphi consensus voting process was used to select a final set of criteria for the checklist. Five groups of stakeholders participated in the Delphi voting process: patients, practitioners, developers, researchers, and policy makers or payers. Each voter was provided with a series of 12 half-page summaries on the quality dimensions (e.g. theoretical rational, evidence) plus the dimension specific criteria for voting. For more detailed information on the quality dimensions, the IPDAS Collaboration’s 12-chapter 2005 Original Background Document was provided. Each voter was asked to rate the importance of each criterion on a 9-point scale. More than 100 stakeholders from 14 countries participated in the first phase of voting; in the second phase, stakeholders were provided summary ratings for each criterion from the first round’s results and asked to re-rate their importance on the 9-point scale. Criteria with median voting scores of 7 or higher on the 9-point scale were retained in the final IPDAS checklist. More details of the voting process and results can be found at the IPDAS website (http://ipdas.ohri.ca).

The final IPDAS Checklist included 74 criteria from 11 of the 12 quality dimensions. Criteria from the quality dimension about “addressing patient stories” did not reach the median score threshold, largely due to uncertainty about the potential benefits and biasing effects of stories in patient decision aids. However, these criteria were added to the checklist as additional criteria to consider when stories are used in patient decision aids. A shorter version of the checklist, limited to those criteria receiving ratings of 9, is used to rate the patient decision aids that are included in the Ottawa A to Z Decision Aid Inventory (http://decisionaid.ohri.ca).

### 2006-2009: Developing the IPDAS instrument

The IPDAS Checklist provides broad assessments of the quality of a patient decision aid across the 12 quality dimensions. But it does not provide precise, quantitative judgments of the decision aid’s quality at criterion (item), dimension, or global levels. To address this concern, the IPDAS Collaboration undertook a project to develop, validate, and report the inter-rater reliability of a measurement instrument designed for quantitatively assessing the quality of patient decision aids—that is, the IPDAS instrument or IPDASi [10].

Criterion-items on the original IPDAS Checklist were refined, items were removed if they did not apply to all decision aids, and the items from the “balancing the presentation of options” dimension were combined with those from the “providing information” dimension. A 4-point rating scale was adopted for each criterion-item, with the following response options: strongly agree, agree, disagree, and strongly disagree. The refinement and confirmation steps yielded 47 items representing 10 dimensions. From there, a validation study was conducted in which the inter-rater reliability of responses on these 47 criterion-items was assessed, using 15 patient decision aids from major producers plus 66 decision aids randomly selected from the Cochrane inventory that is maintained by the Ottawa Patient Decision Aids Group. A short version of the IPDASi was produced, comprising 19 items across 8 of the original dimensions from the checklist [10].

### 2009-2013: Agreeing on minimal standards for certifying patient decision aids

Recognizing that certification of patient decision aids is becoming a priority of health systems in several countries, the IPDAS Collaboration undertook the challenge of identifying a *minimal set of standards* that could be used to certify the quality of a patient decision aid [11]. A modified Delphi process was used to assess each IPDASi criterion on the basis of the potential for risk or harmful bias to the patient’s decision making if the criterion were not present or of low quality in a decision aid. One hundred and one individuals with experience in the field of shared decision making and decision aids voted in the first round, and 87 voted in a second round. The initial set of 47 items from the IPDASi was reduced to 44 items.

A panel of 11 experts was established to reach consensus on minimal criteria based on: a) ratings for the 47 IPDASi items from a modified 2-stage Delphi process; b) qualitative feedback from voters on each of the 47 items in this Delphi process; c) original IPDAS consensus process scores; and d) feedback from 4 trained raters. The expert panel grouped the criteria-items into three broad categories: qualifying criteria, certification criteria, and quality criteria. Six “qualifying criteria” were identified as essential for a tool to be considered a patient decision aid; a tool would not be considered a patient decision aid unless all criteria were met. Six additional criteria were identified as “certification criteria” plus an extra 4 certifying criteria if the patient decision aid is about screening. These criteria were scored on a 1-4 scale (1= strongly disagree and 4 = strong agree); a decision aid must have a score of 3 or higher on each certification criterion to reach certification standards. Finally, a large group of criteria were identified as “quality criteria”, and included those items that were not essential for reducing harms to patients when using decision aids. No threshold is offered for the quality criteria, which are scored on the same 1-4 scale.

### 2011-2013: Updating the evidence underlying the IPDAS checklist

In 2009, the IPDAS Collaboration argued that new concepts and empirical evidence had accumulated since 2005, and consequently there was a need to update the 2005 Original IPDAS Background Document. The IPDAS Background Document Updating Group was charged with this effort.

#### Strategy

The IPDAS Background Document Updating Group consisted of 12 chapter-writing teams (i.e., one team for each of the original 12 quality dimensions). Volunteers who were interested in serving as team leaders, co-leaders, and/or members were identified using several strategies. These included postings on the listserves “Shared-L” and “SMDM-L”, advertisement at the 2009 International Shared Decision Making Conference in Boston, Massachusetts, review of the roster of participants on the 2005 Original IPDAS Background Document, and informal networking among the participants.

Each chapter-writing team leader was identified from senior individuals who had indicated an interest in serving as a team leader, and had conducted relevant research in the area addressed by the specific chapter. Each confirmed team leader was provided with: a) an outline of the updating processes (see below) that their team should follow; b) lists of the names of potential co-leaders and team members who had volunteered through the recruitment strategies described above; and c) the names and contact information of any individuals—including any of those who had co-authored the original 2005 Background Document chapters—who had not directly volunteered but were experts in the field and who might be interested in being involved.

Team leaders selected their co-leaders, and then together the leaders and co-leaders selected the members for their teams. In doing so, they were instructed to consider the diversity of their team (e.g., a mix of basic scientists, decision aid developers, and clinicians), and the importance of international representation on the teams. Final decisions about team membership were made by the individual leaders and co-leaders. Taken together, these teams involved 92 co-authors from 9 countries.

#### Updating processes

Each of the 12 chapter-writing teams was charged with creating an updated chapter consisting of 7 major sections:

1. Current authors and affiliations

2. A chapter summary

3. An updated definition (conceptual/operational) of the quality dimension

4. An updated theoretical rationale for inclusion of the quality dimension

5. An updated evidence base underlying the quality dimension

6. Updated references

7. Appendices, including supporting materials (if needed), and the relevant 2005 Original Background Document Chapter.

Sections 3, 4, and 5 required the most work on the part of a writing team. Within each of these sections, a team’s search, retrieval, and appraisal of the relevant theoretical and empirical literature focused primarily on: a) high-quality publications that are squarely in the field of *patients' decision aids / patients' decision making*; and b) high-quality publications that are clearly in the larger *field of health care in general*. Within each of these sections, a team could also identify particularly important relevant publications in *other non-health-related fields* (e.g., psychology, business, adult education); the points raised by these publications could then be outlined in the "emerging issues / future research" sub-sections of the new chapter in the updated Background Document.

Each team followed established writing guidelines and used a common writing format, as they considered, summarized, and presented the theoretical and evidentiary literature relevant to the quality dimension addressed by their chapter. However, each writing team necessarily worked out its own procedures for dividing up the team’s work, circulating initial drafts among its members, resolving points of discussion via e-mail and/or conference call, and preparing the final submitted version of their updated chapter.

These updating efforts resulted in the IPDAS Collaboration’s new 12-chapter “2012 Updated Background Document” [1]; it can be found at the IPDAS collaboration website (http://ipdas.ohri.ca/resources.html).

## This set of manuscripts

This supplement to *BMC Medical Informatics and Decision Making* builds on the work reported in the 2012 Updated Background Document. Here, a set of 12 manuscripts report—in greater depth than was possible in the 2012 Updated Background Document—the relevant definitions, theoretical justifications, conceptual arguments, evidence-base, and emerging issues relevant to each of the IPDAS Collaboration’s quality standards. A thirteenth manuscript moves beyond the mandate of the 2012 Updated Background Document, and addresses the current challenges inherent in efforts to introduce patient decision aids into routine clinical practice. A total of 102 authors from 10 countries participated in writing these manuscripts. All manuscripts were peer-reviewed as part of the publication process.

### Manuscript highlights

• Angela Coulter and colleagues observe that publications about patient decision aids provide limited detailed information about the processes by which the aids are developed [12]. They present a comprehensive model of the development process, including specific phases of development and a template for reporting key elements in the development process.

• A team led by Michael Barry addresses the issue of disclosing conflicts of interest, emphasizing that disclosures be reported prominently and in plain language [13]. In addition to reporting sources of funding for development and distribution, and whether funders, authors, or their affiliations stand to gain or lose by the choices made by patients using the aids, they propose a third criterion which substantially raises the bar for developers and distributors of patient decision aids. They argue that no funding for the development or exclusive distribution of the decision aid be received from commercial, for-profit entities that stand to benefit from the tests or treatments included as options in the aid.

• Deb Feldman-Stewart and colleagues offer a broad definition of the kinds of information patients need to make informed decisions using patient decision aids [14]. They recommend that investigators drill down to the details related to patients’ information needs and use systematic quantitative study designs to identify the prevalence and extent of variability in these needs.

• A team led by Victor Montori expands the previous chapter on using up-to-date scientific evidence to include evidence that is comprehensive and critically appraised [15]. They further note the importance of communicating the degree of confidence in the evidence, drawing on the approach used by Grading of Recommendations Assessment, Development and Evaluation (GRADE) system.

• Purva Abhyankar and colleagues address the challenging issue of balancing the presentation of information and options in patient decision aids [16]. They provide evidence supporting the use of side-by-side presentation formats for the information about the options being presented in a patient decision aid. They also raise the provocative question of whether patient decision aids should ever be used to nudge patients toward certain options.

• Lyndal Trevena, Brian Zikmund-Fisher and colleagues move beyond the background document on communicating probabilities to summarize the state of the science in presenting quantitative information about decision outcomes in patient decision aids [17]. In doing so, they have developed a risk communication primer for decision aid developers, and a major resource for the field in general, addressing 11 key issues when presenting quantitative information.

• Theoretical and methodological issues in the use of values clarification methods in patient decision aids are addressed by the team headed by Angela Fagerlin and Michael Pignone [18]. This group argues that values clarification methods can focus on attributes of options, options as whole entities, and the decision context. They further note a number of promising decision making theories that could be used to guide design and evaluation of values clarification methods.

• Hilary Bekker heads a group of investigators considering the role of personal stories in patient decision aids, addressing the question about whether or not stories make aids more effective [19]. They emphasize that developers should be explicit about the purpose of patient stories in their aids and that the field could benefit from a taxonomy of stories in decision aids. They further note the tremendous scope for research on the role of stories in decision making and behavioral change, including identifying active components of stories and how they facilitate or bias decision making.

• A team headed by Kirsten McCaffery and Stacey Sheridan tackle the rapidly evolving field of health literacy, noting new definitions that recognize multiple levels of health literacy beyond reading comprehension [20]. They note that patient decision aids are rarely developed with lower literacy populations in mind, and measures of health literacy and readability of patient decision aids are rarely reported. They further identify specific research gaps in the field (e.g., the role of health literacy in the process of values clarification) to guide patient decision aid developers.

• Dawn Stacey and colleagues review theoretical and empirical developments related to guidance (i.e., explicit elements in patient decision aids that facilitate self-directed decision making) and coaching (i.e., the use of trained individuals who support decision making) with patient decision aids [21]. Among the randomized trials they found that coaching provided with a patient decision aid was associated with improved outcomes; no trials were available on elements of guidance. They advocate for randomized trials to compare the effectiveness of coaching used with and without decision aids, and decision aids used with and without coaches.

• Constructs and measurement instruments for establishing the effectiveness of patient decision aids are addressed by a team led by Karen Sepucha and Richard Thomson [22]. They distinguish the quality of the decision-making process and the quality of the choice, and find strong evidence that patient decision aids enhance both process and quality decisions for patients. They further note the need for consensus around standardized measures for establishing the effectiveness of patient decision aids.

• Aubri Hoffman heads a group looking at the rapidly evolving field of patient decision aids delivered on the Internet [23]. They identify different characteristics of patient decision aids delivered on the Internet, ranging from aids developed for other formats and placed on the Internet to those specifically designed and tested for use on the Internet. Currently, few randomized trials on Internet-delivered patient decision aids have been conducted, and this group offers a rich agenda for basic and applied research in this area.

• The final manuscript is this supplement addresses the challenges of implementing patient decision aids in routine clinical settings. Elwyn and colleagues report the findings from a systematic review about the implementation of patient decision aids (they use the term “patient decision support interventions”) in clinical settings, noting barriers related to implementation including apparent indifference on the part of health care professionals regarding use of these tools [24]. They further acknowledge that it is too early to make firm recommendations about best strategies for implementation of patient decision aids in routine clinical settings.

### A note about funding

The IPDAS Collaboration’s 2012 Updated Background Document was prepared without centralized financial support. Individual chapter leads/co-leads/members indirectly were supported by their affiliation sites while they worked as volunteers on this project, and administrative support for the entire update process was provided as “gifts in kind”.

Furthermore, no centralized support was sought or provided for creation of the manuscripts included in this *BMC Medical Informatics and Decision Making* supplement. The individuals involved in writing these papers used a variety of funding sources, including support from their institutions. The Informed Medical Decisions Foundation supported publication of this supplement by covering about half of the publication fees.

## In conclusion

This set of 13 manuscripts highlight a wide range of fundamental and applied research issues that are highly relevant to the creation, evaluation, and implementation of patient decision aids. In the face of this multi-faceted argument, there are differing opinions about which are the most pressing research priorities in this field of inquiry. We think that neither the "let's-get-all-the-basic-research-questions-dealt-with-before-we-go-any-further" perspective nor the "we-know-enough-so-let's-put-all-our-resources-into-full-bore-implementation" perspective is of greater importance. We think that, ultimately, patients, practitioners, students, new investigators in the field, policy-makers, and health care leaders will most benefit if both streams of investigations— those motivated by fundamental questions and those focusing on implementation problems—are able to proceed in parallel, in a collegial, interdisciplinary manner.

## List of abbreviations used

IPDAS: International Patient Decision Aid Standards; SDM: Shared decision making; IPDASi: IPDAS instrument

## Competing interests

Robert J. Volk and Glyn Elwyn have received research funding, travel support and honoraria from the Informed Medical Decisions Foundation, a not-for-profit (501 (c) 3) private foundation (http://www.informedmedicaldecisions.org). The Foundation develops content for patient education programs. The Foundation has an arrangement with a for-profit company, Health Dialog, to co-produce these programs. The programs are used as part of the decision support and disease management services Health Dialog provides to consumers through health care organizations and employers.

Hilary Llewellyn-Thomas and Dawn Stacey have no competing interests to declare.

## Declarations

Publication of this supplement was partially funded by an unrestricted grant from the Informed Medical Decisions Foundation. The remainder costs were covered by Robert J. Volk. Administrative and editorial support for this supplement was also provided by The University of Texas MD Anderson Cancer Center.

This article has been published as part of *BMC Medical Informatics and Decision Making* Volume 13 Supplement 2, 2013: The International Patient Decision Aid Standards (IPDAS) Collaboration’s Quality Dimensions: Theoretical Rationales, Current Evidence, and Emerging Issues. The full contents of the supplement are available online at http://www.biomedcentral.com/bmcmedinformdecismak/supplements/13/S2.
